# Analysis of Hot Springs, Arkansas

**Published:** 1869-03-15

**Authors:** E. Hills Larkin

**Affiliations:** St. Louis, Mo.


### CHICAGO MEDICAL JOURNAL.

VOL. XXVI.—MARCH 15, 1869.—No. 6.

### ORIGINAL COMMUNICATIONS.

### *Analysis of Hot Springs, Arkansas.*

MADE BY E. HILLS LARKIN, OF ST. LOUIS, MO., JUNE 1, 1859.

NUMERICAL CONSTITUENTS—8½ PER GALLON.

PERCENTAGE.

| | |
|---|---|
| Silicic Acid | 24.74 |
| Sesque-Oxide of Iron | 1.21 |
| Alumna | 5.15 |
| Lime | 28.83 |
| Magnesia | .73 |
| Chlorine | .67 |
| Carbonic Acid | 21.36 |
| Organic Matter | 8.31 |
| Water | 1.72 |
| Sulphuric Acid | 4.49 |
| Potassa | 1.46 |
| Soda | 2.01 |
| Total | 100.00 |

To the above analysis, Bromine and Iodine should be added; for in 1859 Dr. David Dale Owen extracted the solid residue from 2,000 grammes, with alcohol; evaporated this to dryness at a low temperature, and tested it with proto-chloride of palladium. The watery solution was slightly tinged yellowish-brown, indicative of a trace of sodium; "and," says Dr. Owen, "if larger quantities of the water were operated on, the Iodine and Bromine could, in all probability, be distinctly brought out."

I have noticed, briefly, the constituents of these valuable waters, as given by able minds, and will now as briefly notice their action upon the diseased system:

It has been my province, for the past two years, to treat, with the assistance of these issues, many forms of chronic disease, such as rheumatism, gout, stiff-joints, contraction of the muscles, and skin-diseases as a class; scrofula, enlargement of the glands, general debility, spinal diseases, neuralgia, nervous affections, dyspepsia, nervous and mucous paralysis, in its varied forms, uterine diseases as a class, mercurial, mercurio-syphilitic, and all forms of syphilitic disease. Many diseases, which are too numerous to mention, are likewise benefited by the judicious use of the waters; but the most marked improvement is shown in rheumatism, gout, skin-diseases, neuralgias, uterine affections, mercurial and syphilitic.

In many cases referred to, their effects are truly astonishing.

The copious diaphoresis which the hot-bath establishes opens in itself a main channel for the expulsion of principles injurious to health, which is made manifest through the pores of the skin, and by the peculiar odor; it alike arises from the internal use of the water, in a diminished degree. The impression produced by the hot-douche, as is referred to, is indeed powerful, arousing into action sluggish and torpid secretions; hence the languid circulation is purified of morbific matter; renewed vigor and healthful action are thus given to the absorbents, lymphatics, and to the excretory apparatus, a combined effect which no medicine is capable of accomplishing.

The carbonates of the alkalies and alkaline-earths, by their reaction cannot be without their therapeutic effects, as is shown by observation, over the diseases mentioned.

The chlorides and sulphates, although small, have their effects upon the physical economy.

The exhilarating effects upon the system of the large quantities of free carbonic-acid, which the water contains, proves an auxilliary in sustaining the system during the change of tissue, and of the blood, which is consequent upon their constant and continued use.

In the above diseases tonics and alteratives are demanded; hence the potency of the waters will seem tangible to the minds of the profession, when conjoined with proper treatment and sound advice.

The mild latitude and even temperature of this section of Arkansas make it a truly desirable resort for invalids, throughout the entire year. For the past two years I have watched closely the thermometer, and find, in mid-summer, the hottest day reached only 96° Fahr., while, this winter, our coldest day did not fall below 28° Fahr. (above zero).

Daily stages reach this point from the Capital of the State. P. N. Ellsworth.

Hot Springs, Ark., Feb. 17, 1867.

